# The Effect of Polyphenols on Pomegranate Fruit Susceptibility to *Pilidiella granati* Provides Insights into Disease Tolerance Mechanisms

**DOI:** 10.3390/molecules25030515

**Published:** 2020-01-24

**Authors:** Annamaria Mincuzzi, Antonio Ippolito, Virginia Brighenti, Lucia Marchetti, Stefania Benvenuti, Angela Ligorio, Federica Pellati, Simona Marianna Sanzani

**Affiliations:** 1 Dipartimento di Scienze del Suolo, della Pianta e degli Alimenti, Università degli Studi di Bari Aldo Moro, Via Amendola 165/A, 70126 Bari, Italy; annamaria.mincuzzi@uniba.it (A.M.); antonio.ippolito@uniba.it (A.I.); angela.ligorio@ipsp.cnr.it (A.L.); 2 Dipartimento di Scienze della Vita, Università degli Studi di Modena e Reggio Emilia, Via G. Campi 103, 41125 Modena, Italy; virginia.brighenti@unimore.it (V.B.); lucia.marchetti@unimore.it (L.M.); stefania.benvenuti@unimore.it (S.B.); 3 Scuola di Dottorato di Ricerca in Medicina Clinica e Sperimentale (CEM), Università degli Studi di Modena e Reggio Emilia, Via G. Campi 103, 41125 Modena, Italy; 4 Istituto per la Protezione Sostenibile delle Piante (IPSP), Sede Secondaria di Bari, Consiglio Nazionale delle Ricerche, Via Amendola 122/D, 70126 Bari, Italy; 5 CIHEAM-Bari, Via Ceglie 9, 70010 Valenzano (BA), Italy

**Keywords:** *Punica granatum*, pomegranate, polyphenols, *Pilidiella granati*, antifungal activity, qPCR, HPLC, MS

## Abstract

*Pilidiella granati*, also known as *Coniella granati*, is the etiological agent of pomegranate fruit dry rot. This fungal pathogen is also well-known as responsible for both plant collar rot and leaf spot. Because of its aggressiveness and the worldwide diffusion of pomegranate crops, the selection of cultivars less susceptible to this pathogen might represent an interesting preventive control measure. In the present investigation, the role of polyphenols in the susceptibility to *P. granati* of the two royalties-free pomegranate cultivars Wonderful and Mollar de Elche was compared. Pomegranate fruit were artificially inoculated and lesion diameters were monitored. Furthermore, pathogen DNA was quantified at 12–72 h post-inoculation within fruit rind by a real time PCR assay setup herein, and host total RNA was used in expression assays of genes involved in host-pathogen interaction. Similarly, protein extracts were employed to assess the specific activity of enzymes implicated in defense mechanisms. Pomegranate phenolic compounds were evaluated by HPLC-ESI-MS and MS^2^. All these data highlighted ‘Wonderful’ as less susceptible to *P. granati* than ‘Mollar de Elche’. In the first cultivar, the fungal growth seemed controlled by the activation of the phenylpropanoid pathway, the production of ROS, and the alteration of fungal cell wall. Furthermore, antifungal compounds seemed to accumulate in ‘Wonderful’ fruit following inoculation. These data suggest that pomegranate polyphenols have a protective effect against *P. granati* infection and their content might represent a relevant parameter in the selection of the most suitable cultivars to reduce the economic losses caused by this pathogen.

## 1. Introduction

Pomegranate (*Punica granatum* L.) is one of the emerging crops worldwide. It originated in the Transcaucasia-Caspian region and Northern Turkey [1], from where it spread to the Mediterranean basin [2]. However, Spain and Italy are the main European producers. In 2012, in the Iberian Peninsula, 3344 ha of pomegranate were cultivated and 45,000 t produced; two years later, a 15–20% increment both in production and in orchards’ dimensions was recorded [3]. In Italy, about 4000 ha of pomegranates were cropped in 2017 and more than 60,000 t produced [4]. The best Italian producers are the Apulia and Sicily regions. According to the most recent available data, Apulia recorded a 70–80% increase in sold shrubs belonging to cultivars Wonderful and Akko, which are royalties-free [4].

The great appreciation of consumers for pomegranate is mainly due to its organoleptic and nutraceutical properties. Indeed, both the fruit (peel, seeds, and arils) and the plant (cork and roots) are rich in bioactive compounds, whose presence has encouraged the consumption and the demand of pomegranates as fresh and by-products. Particularly, the rind is rich in phenolics (including flavonoids, ellagitannins, and proanthocyanidins), minerals, and complex polysaccharides [5,6]. Some of these bioactive compounds, such as phenolics, exist also in the arils, which are constituted by water, sugars (fructose and glucose), pectin, and organic acids (ascorbic, citric, malic). All these substances make pomegranate and the by-products useful in the prevention and control of human diseases, because of their antioxidant and anti-inflammatory properties. In addition, the richness in chemopreventive compounds might have antiproliferative, antimicrobial, and neuroprotective effects [7,8].

The increasing market demand and the difficulty in storing the fruit for a long period of time have boosted scientific research on the characterization and control of the main pomegranate pathogens and the overall improvement of the fruit postharvest life. One of the most important causal agents of fruit rot is *Pilidiella granati* (Saccardo), also known as *Coniella granati* (Sacc.) Petr. and Syd. Indeed, *P. granati* is the etiological agent of plant collar rot and fruit rot [9,10,11,12,13], as well as of leaf spot [14]. This host-specific pathogen can cause shrubs wilts [15,16,17] and important (10–30%) postharvest yield losses of fruit [9,16,17,18]. Fruit symptoms consist of circular brownish-yellow lesions of the rind that start in the crown area, and then spread to the entire fruit, causing progressive deliquescence and darkening of mesocarp, endocarp, and arils [15,19]. The growth and evasion of this fungus are simultaneous and end up with the development of mycelium and pycnidia on the rotted rind. The knowledge about the epidemiology of this fungus is quite limited [20]. However, this thermophilic pathogen, which prefers temperatures between 25 and 30 °C and 80% relative humidity (RH) [17,21], probably infects fruit during blooming or through the crown of young fruit, and remains latent until fruit ripening or breaks out during the postharvest stage. *P. granati* has a significant ability to overwinter in mummies and littered branches; this characteristic assures inoculum availability in every season [17,18,22]. Since a different susceptibility of various pomegranate cultivars to this fungus has been reported [20], the main aim of this research was to evaluate the effect of polyphenols from two commercially diffused pomegranate cultivars (Mollar de Elche and Wonderful) with putative different susceptibility to the disease [23], by traditional, molecular, and biochemical approaches, in order to have an overview of the host-pathogen interaction and to define more proper control strategies.

## 2. Results

### 2.1. Susceptibility Assays

**Figure 1:**
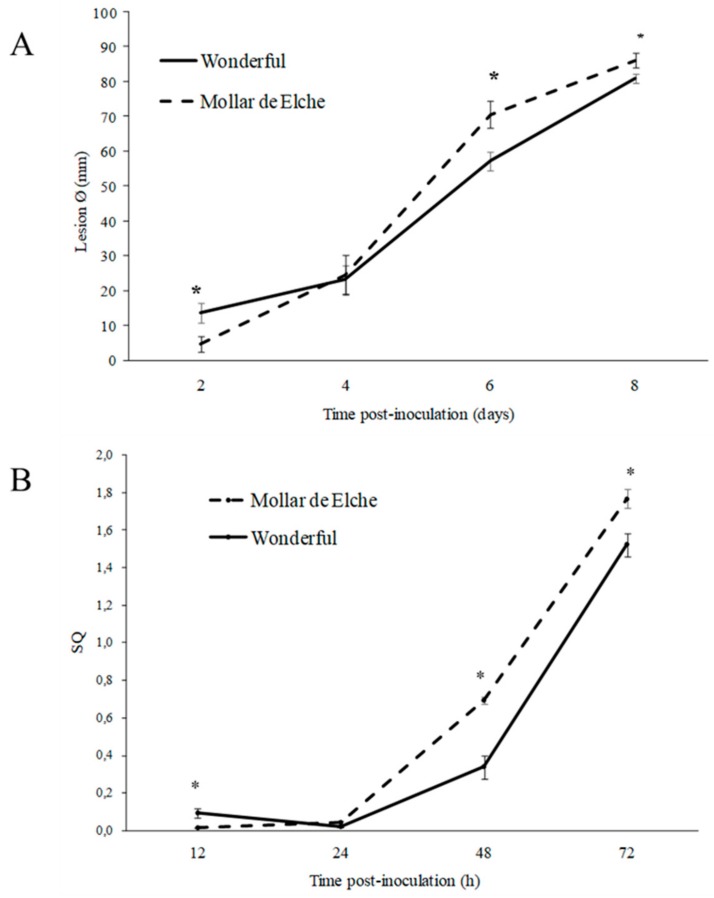
Growth curve of *P. granati* artificial inoculum on the two pomegranate cultivars Wonderful and Mollar de Elche: (**A**) lesion Ø (mm) at 2–8 days post-inoculation; (**B**) SQ of pathogen DNA in qPCR reaction, coming from inoculated fruit sampled at 12–72 h post-inoculation. Data represent the mean of three values ± standard error of mean (SEM). Time points with an asterisk are statistically different according to Duncan’s Multiple Range Test (DMRT, * *p* < 0.05).

To compare the susceptibility of pomegranate cultivars Mollar de Elche and Wonderful to *P. granati*, the disease incidence and severity in artificially inoculated fruit, and the amount of pathogen DNA within the overall host tissues were analyzed. At two days post-inoculation, all wounds were infected (data not shown), and the mean lesion diameter on ‘Wonderful’ and ‘Mollar de Elche’ pomegranates was 13.8 and 4.8 mm, respectively (Figure 1A). At four days post-inoculation, the mean lesion diameter of both cultivars was about 24 mm, but, from this time point on, ‘Mollar de Elche’ pomegranates showed a greater mean lesion diameter than ‘Wonderful’ ones. Particularly, at six days post-inoculation, ‘Mollar de Elche’ and ‘Wonderful’ showed a mean lesion diameter of 70.7 mm and 57.3 mm, respectively.

Concerning the monitoring of *P. granati* DNA in the fruit tissues of the two cultivars, a quantitative real time PCR (qPCR) assay was set up. The high conservation of ITS region within the same fungal species and variability among the different species of the genus allowed designing the specific primer pair PILF (5′-TCATCGAATCTTTGAACGCACA-3′)/PILR (5′-CTCAGAGTCTTAGCGAGCCC-3′), which showed a distinct band of 161 bp for *P. granati*, whereas the other pomegranate pathogenic fungal genera did not show any amplification (data not shown). Furthermore, the standard curve (y = −3.579x + 18.382), obtained testing 10-fold dilutions of *P. granati* DNA, showed a strong linear relationship (R^2^ = 1) and an efficiency of 90.3% (included in the optimal range 90–110%). The system proved to be sensitive enough to efficiently amplify up to 390 fg of target DNA. Comparable results were obtained with the pomegranate specific qPCR assay based on the elongation factor (EF-1α) gene (Table 1); in fact, the standard curve had an equation of y = −3.548x + 22.422, a R^2^ = 0.998, and a reaction efficiency of 91.4%. Comparing the abundance of *P. granati* DNA in inoculated fruit of ‘Wonderful’ and ‘Mollar de Elche’, a trend very similar to the one observed for disease severity was recorded (Figure 1B). To take into account variations in sample size and quality, the concentration of *P. granati* DNA was expressed as standard quantity (SQ). Initially, at 12 h from inoculation, there was a prominence of *P. granati* DNA in ‘Wonderful’ as compared to the ‘Mollar de Elche’ fruit, whereas, at 24 h, it was comparable in both cultivars; then, a higher SQ of DNA was recorded in ‘Mollar de Elche’ fruit as compared to ‘Wonderful’ ones (Figure 1B). By the end of incubation, average values of 1.5 and 1.8 SQ were recorded for ‘Wonderful’ and ‘Mollar de Elche’, respectively. Wounded negative controls of both cultivars did not produce any increase in fluorescence (data not shown).

### 2.2. Gene Expression Assays

In order to better understand the putative differences between the two pomegranate cultivars in terms of host-pathogen interaction, the expression of genes known to be involved in host defensive mechanisms, namely chitinase, phenylalanine ammonia lyase (PAL), and peroxidase, was evaluated. EF-1α was used as the housekeeping gene. To this aim, specific primer pairs were designed herein. Linear equations, R^2^ values, and reaction efficiencies are reported in Table 1.

**Figure 2:**
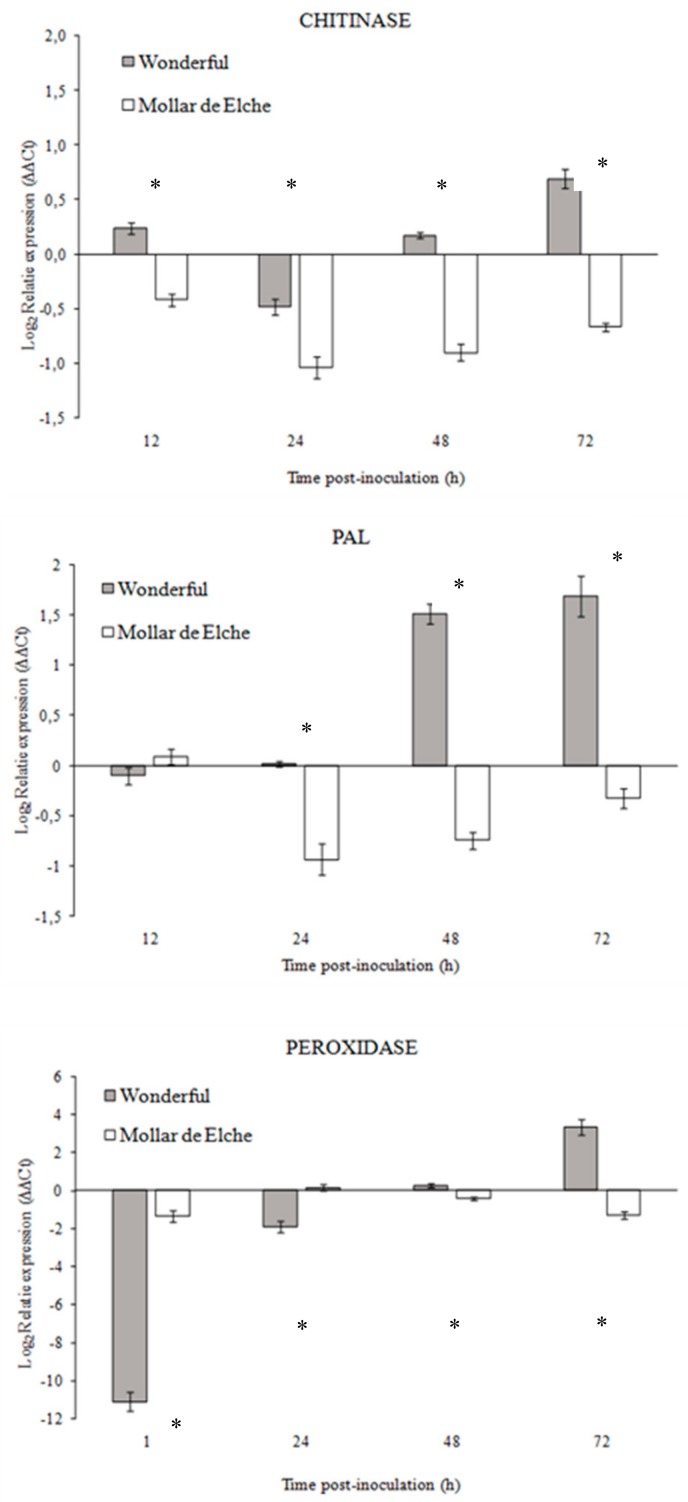
Relative expression (log_2_ transformed) of chitinase, PAL, and peroxidase genes in ‘Wonderful’ and ‘Mollar de Elche’ rinds inoculated by *P. granati,* as compared to wounded non-inoculated fruit, at 12–72 h post-inoculation. Data were analyzed using the 2^−∆∆Ct^ method and normalized using the housekeeping gene EF-1α. They represent the mean of three values ± SEM. Time points with an asterisk are statistically different according to DMRT (* *p* < 0.05).

The Ct values and the RNA concentrations were linearly correlated for every examined gene. Similar and optimal reaction efficiencies were assessed for the housekeeping gene and the three target genes. The presence of a unique melting peak for each gene in the melting curve analysis indicated that all primer pairs amplified a single product with a distinct melting temperature (data not shown). Negative control samples did not display any fluorescent signal, proving both the absence of contaminations in the reaction mixtures and the complete removal of DNA traces. As the cDNA synthesized from 100 ng of total RNA was efficiently amplified, this concentration was used in the subsequent qPCR reactions. Relative expression patterns of the selected genes involved in defense response in pomegranate rind tissues were evaluated at four different time points post-inoculation (12, 24, 48, and 72 h) (Figure 2). Chitinase, PAL, and peroxidase genes proved to be up-regulated in ‘Wonderful’ tissues since 48 h post-inoculation, while they were down-regulated in the ‘Mollar de Elche’ fruit. The peak of induction in ‘Wonderful’ fruit was recorded at 72 h post-inoculation, being low, medium, and high for chitinase, PAL, and peroxidase, respectively (Figure 2).

### 2.3. Enzymatic Assays

**Figure 3:**
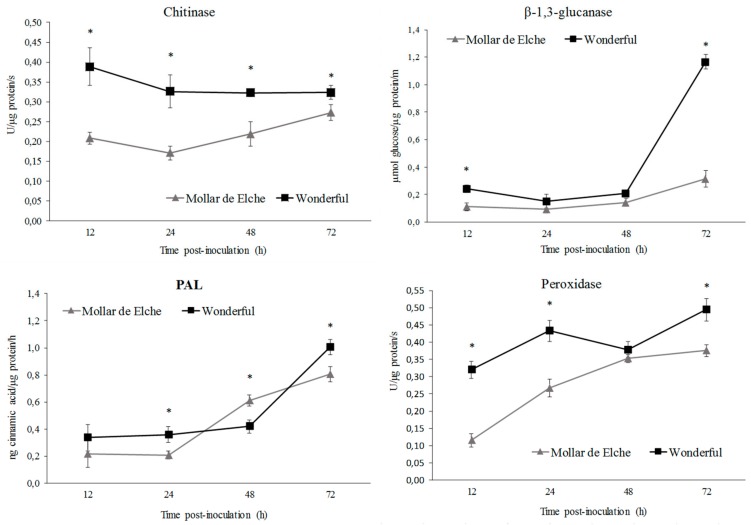
Time course of the activity of the enzymes chitinase, β-1,3-glucanase, PAL, and peroxidase in extracts from ‘Wonderful’ and ‘Mollar de Elche’ rinds inoculated by *P. granati*. Each unit of measurement is calculated on a total protein basis. Data are the mean of three experiments ± SEM. Time points with an asterisk are statistically different according to DMRT (* *p* < 0.05).

As further analysis, the enzymatic activity of chitinase, peroxidase, and PAL, plus β-1,3-glucanase, was evaluated. These data essentially confirmed the previous ones. Overall, the enzymatic activity in ‘Wonderful’ tissues was higher than in ‘Mollar de Elche’ ones (Figure 3). In particular, considering the specific activity of chitinase, the highest differences between both cultivars were recorded at 12 and 24 h. Then, it remained constant in ‘Wonderful’, while it increased in ‘Mollar de Elche’, thus reducing the differences in activity at the following two time-points (Figure 3). The β-1,3-glucanase showed the strongest increase (>5-fold) in activity at 72 h in the ‘Wonderful’ fruit, as compared to the ‘Mollar de Elche’ ones, whereas in previous time points it was comparable for the two cultivars (Figure 3). Concerning PAL specific activity, both ‘Wonderful’ and ‘Mollar de Elche’ fruit had similar initial values, ranging between 0.20 and 0.30 ng/µg/h of cinnamic acid on a total protein basis at 12 h post-inoculation (Figure 3). Then, starting from 48 and 72 h, the specific activity of PAL increased in cultivars Mollar de Elche and Wonderful, respectively, reaching in the latter case a peak of 1.1 ng/µg/h of cinnamic acid on a total protein basis. A similar trend to that of chitinase was observed for the specific activity of peroxidase (Figure 3): indeed, it was higher in ‘Wonderful’ as compared to ‘Mollar de Elche’ fruit, having a peak of 0.5 U/μg/s on a total protein basis at 72 h. However, the strongest differences between the two cultivars were observed at 12 and 24 h from inoculation. In particular, an up to 3-fold induction was observed in the ‘Wonderful’ fruit as compared to the ‘Mollar de Elche’ ones.

### 2.4. Chemical Analysis of Phenolics

**Figure 4:**
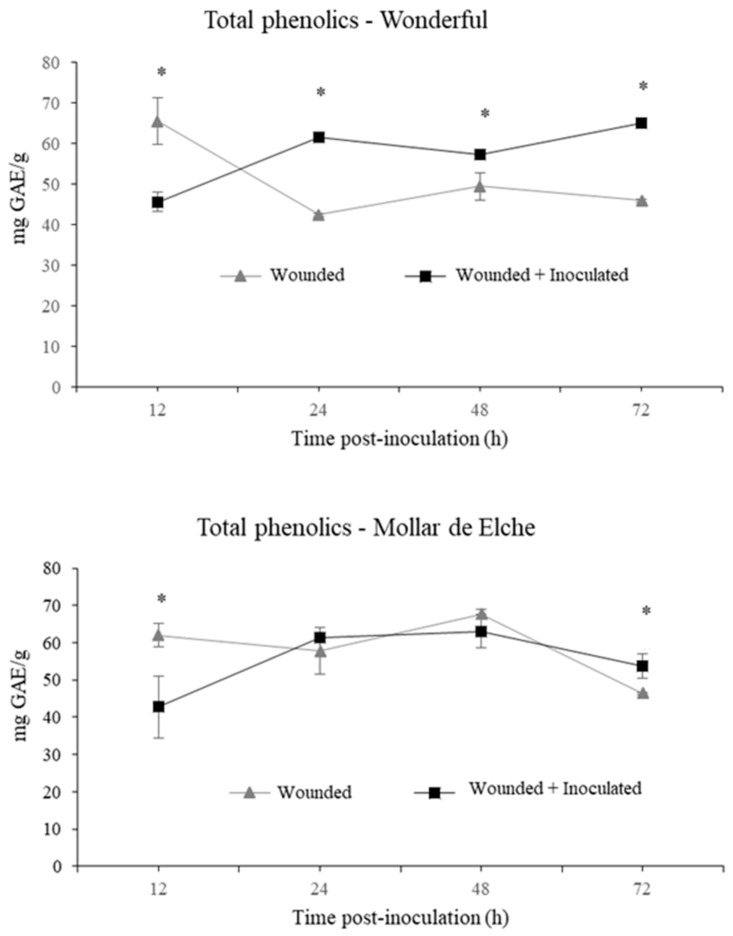
Total phenolics (mg/g GAE) in rinds of ‘Wonderful’ and ‘Mollar de Elche’ fruit inoculated by *P.* *granati*, as compared to the wounded ones at 12–72 h post-inoculation. Data are the mean of three experiments ± SEM. Time points with an asterisk are statistically different according to DMRT (* *p* < 0.05).

By comparing the quantitative data of the content of total phenolics (Figure 4), the cultivar Mollar de Elche showed generally a lower increase of these compounds after the inoculation by *P. granati* than the ‘Wonderful’ one. Particularly, the rind of the latter cultivar at 24 h post-inoculation showed a radical increase in their content. At 12 h sampling, the wounded samples had a content of total phenolics of 66 mg/g gallic acid equivalents (GAE), while the inoculated ones at the same sampling time scored 46 mg/g GAE. At 72 h post-inoculation, the wounded and inoculated samples of ‘Wonderful’ displayed a total phenolic content of 46 and 65 mg/g GAE, respectively. As regards cultivar Mollar de Elche, the inoculated samples showed a higher phenolic content than non-inoculated ones only at 72 h (Figure 4).

**Figure 5:**
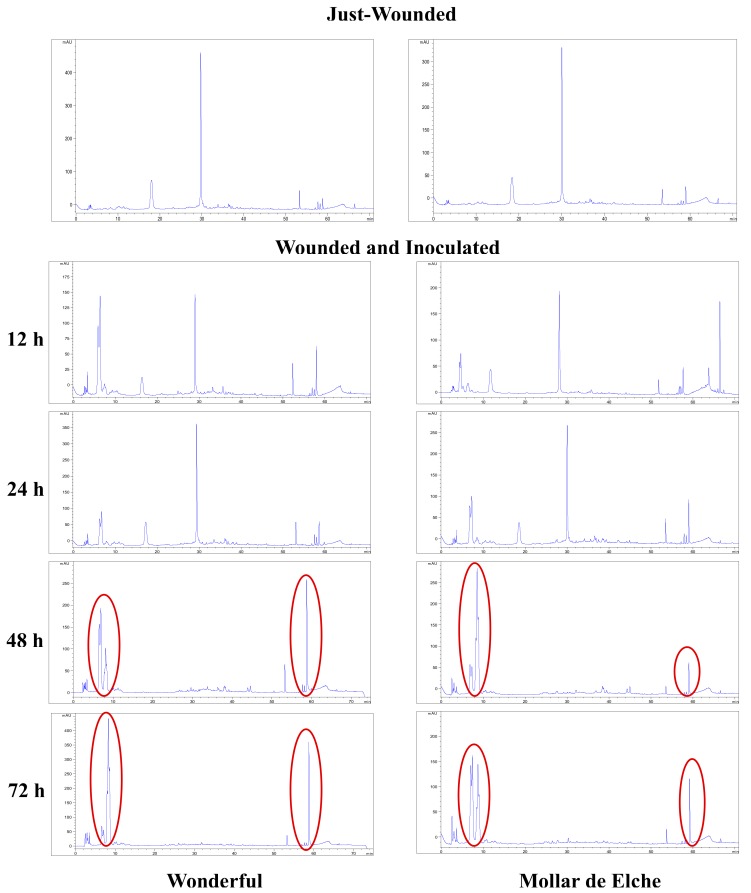
HPLC-UV/DAD chromatograms of ‘Wonderful’ and ‘Mollar de Elche’ rind extracts. Fruits were just-wounded or wounded and inoculated by *P. granati*. Cultivars were compared at 12–72 h post-inoculation. Punicalagin hydrolysis products are circled by red lines.

By focusing on the polyphenol profiles obtained by HPLC-UV/DAD analysis for the ‘Wonderful’ fruit at 12 and 24 h post-inoculation, a difference was observed as compared to the wounded samples and, above all, at the following time points (48 and 72 h post-inoculation) (Figure 5). Indeed, at 48 and 72 h post-inoculation, the samples displayed two significant groups of peaks: the first with a retention time (*t_R_*) between 2 and 8 min and the second from 50 to 60 min. By examining the extracts of the samples corresponding to 48 h post-inoculation by HPLC-ESI-MS and MS^2^ in the negative ion mode, the compounds corresponding to peaks detected in HPLC-UV/DAD were identified (Table 2). Particularly, the first group of peaks corresponded to gallagic acid, hexahydroxydiphenoyl (HHDP)-glucose, and punicalin. The second group of peaks was related to ellagic acid hexoside, ellagic acid pentoside, ellagic acid deoxyhexoside, and ellagic acid. The two peaks eluting at 16–30 min (Figure 5), more evident at 12 and 24 h post-inoculation and present in traces at 48 h, were identified as α- and β-punicalagin (Table 2). A similar trend was observed at 48 and 72 h also for the inoculated samples of the ‘Mollar de Elche’ cultivar, but the areas of the second group of peaks, and thus the concentrations of related compounds, were lower (Figure 5).

## 3. Discussion

Several elements can influence a complex system as that of a host-pathogen interaction. For example, Palou et al. [24] observed that the etiology and incidence of pomegranate postharvest diseases, as the rot caused by *P. granati*, might depend on the environmental characteristics of the growing area, as well as on the conditions in the preharvest, harvest, and postharvest phases. Kahramanoglu et al. [25] stressed the importance of floral morphology and ripening time to explain the susceptibility to heart rot caused by *Alternaria* spp. on different pomegranate cultivars; Herskovitz cultivar was found to be more susceptible than ‘Wonderful’ and less than ‘Akko’. Furthermore, pomegranate fruit, in the same environmental conditions, displayed a different susceptibility to cracking according to the cultivar [26]; this finding is interesting considering that cracks could help pathogen penetration and spreading. Since no or few treatments are allowed on pomegranate in many countries, including Italy, to reduce yield losses caused by biotic agents, researchers have been looking for resistant cultivars for a long time [27,28]. In this context, considering the relevance of the economic losses due to *P. granati* postharvest fruit rots [9,16,17,18], the importance of understanding this host-pathogen interaction is evident in order to select less susceptible cultivars. In the present investigation, the susceptibility to *P. granati* of mature pomegranate fruit of the two commercial cultivars Wonderful and Mollar de Elche from organic agriculture of southern Italy were compared.

Looking for pomegranate genotypes resistant to anthracnose, in a study by Jayalakshmi et al. [29], leaves of various Indian pomegranate cultivars were artificially inoculated by *Colletotrichum gloeosporioides*; none of them appeared resistant, even if this research highlighted differences in susceptibility. Similar results were obtained testing in the field the pathogenicity of both leaves and fruit infected by *P. granati* [30], disclosing some cultivars moderately resistant to leaf spot and dry rot, respectively. Recently, Jabnoun-Khiareddine et al. [20] have evaluated the susceptibility of Tunisian pomegranate cultivars to *P. granati* artificial infections; the authors reported that, although all tested cultivars completely rotted within 11–20 days from inoculation, they showed significant differences in susceptibility. Finally, the cultivar Wonderful and its clones (e.g., ‘Wonderful One‘) and the cultivar Akko have been compared for their susceptibility to other fungal pathogens [31]. ‘Akko’ revealed to be the most and ‘Wonderful’ the least susceptible cultivar, respectively. Although all these studies seem to confirm the existence of significant differences within pomegranate cultivars, a deeper comparison of the responses of two of the most important commercial cultivars, namely Wonderful and Mollar de Elche, to *P. granati* infections is missing and it might be useful in the research of resisting genotypes. 

According to the herein obtained results, *P. granati* caused lesions onto ‘Mollar de Elche’ around 30% more severe than on ‘Wonderful’. This circumstance might be relevant in an integrated approach to prolong storage and shelf-life of pomegranate fruit, which represent a high added-value produce. These findings were confirmed by the quantification by qPCR of fungal DNA within the host DNA, as already described for other host-pathogen interactions [32]. The assay proved to be specific and sensitive enough to detect 390 fg of pathogen DNA; thus, it could be applied to the early detection of *P. granati*, even as latent, incipient, or quiescent infection. Furthermore, SYBR green as fluorescent dye makes the assay cheap and easily applicable to different tissues. Finally, the assay proposed herein could provide a disease monitoring in a couple of hours, through the analysis of flowers, branches, shoots, and mummies, and thus the enrichment of epidemiological knowhow, favoring prevention practices. As such, it might represent an improvement of the detection methods already described in the literature [33].

Given the higher susceptibility of ‘Mollar de Elche’ as compared to ‘Wonderful’ fruit to *P. granati*, a multiple approach was used to investigate the mechanisms involved in this host-pathogen interaction. The differential expression of genes known to be responsible for resistance mechanisms, as well as the activity of related enzymes, was evaluated. Although few pomegranate gene sequences were available at the time in public databases, it was possible to design specific primers for chitinase, PAL, and peroxidase genes. In presence of *P. granati* inoculum, all the tested genes showed a stronger up-regulation in ‘Wonderful’ as compared to ‘Mollar de Elche’. These results suggest the induction of the phenyl-propanoid pathway, as well as the production of ROS and the degradation of the fungal cell walls, as mechanisms putatively involved in the lower susceptibility of this cultivar to the pathogen, as observed also for *Penicillium digitatum*-citrus interaction [34]. These findings were confirmed by the enzymatic assays, which evidenced also the stronger activity of β-1,3-glucanase in ‘Wonderful’ as compared to ‘Mollar de Elche’ fruit. Balaganur [35] evaluated the pomegranate response to *Ceratocystis fimbriata*, obtaining similar results (i.e., the up-regulation of PAL and peroxidase). Peroxidase has been reported to coordinate and organize various defensive barriers against pathogens [36], and PAL has been found to be involved in priming, increasing the capacity to mobilize pathogen- or elicitor-induced cellular defense responses [37]. Both chitinase and β-1,3-glucanase have been identified as pathogenesis-related proteins, coded by host plant as specific response to pathogen attacks, but systemically diffused and related to systemic acquired resistance (SAR), showing a well-known antifungal activity [38]. This theoretical framework became clear observing both the genes over expression and the overall rise of enzymes in the inoculated samples, starting from 24 to 48 h post-inoculation; probably, further time points over 72 h could have provided interesting data, such as major increment in enzyme specific activity. Consequently, the fruit should be able to better defend itself against fungal attacks.

Nevertheless, ‘Mollar de Elche’ fruit might be more susceptible to lytic enzymes of *P. granati*, thus enabling the fungus to trespass the rind and to penetrate into the pomegranate fruit [20]. A further analysis of *Coniella lustricola* genome by Raudabaugh et al. [39] has confirmed the existence of genes involved in lysis. Among them, the presence of genes for aryl sulfotransferase was relevant, since these enzymes proved to be able to degrade toxic phenolic compounds abundant in pomegranate fruit, especially in the rind, as punicalagin [39].

The induction of the phenyl-propanoid pathway was suggested even by the changes in the amount and composition of the total phenolics contained in pomegranate rind, which were visible especially in the inoculated ‘Wonderful’ fruit, showing a significant amount of 65 mg/g GAE at 72 h post-inoculation. In this context, α- and β-punicalagin represent the main ellagitannins of the rind extracts up to 24 h, as described by Brighenti et al. [40]. Starting from 48 h post-inoculation, other phenolic compounds arose in inoculated pomegranates of both cultivars, including gallagic acid, HHDP-glucose and punicalin, which eluted first, followed by ellagic acid hexoside, ellagic acid pentoside, ellagic acid deoxyhexoside and ellagic acid, which eluted later in the chromatographic run [40]. In general, phenolic compounds were more abundant in the inoculated samples of ‘Wonderful’ than in the ‘Mollar de Elche’ ones, particularly at 72 h post-inoculation. In addition, a positive feedback mechanism might be speculated, in which polyphenols degradation products stimulate their production, because they are involved in plant responses to stress conditions [41]. Similarly, the existence of the enzyme ellagitannase, which hydrolyzes ellagitannins into ellagic acid, has been previously observed in relation with *Aspergillus niger* both *in vitro* [42] and in rind extracts [43]. According to Fischer et al. [44], punicalagin, the main ellagitannin present in pomegranate rind, is probably the precursor of punicalin. Gallagic acid is an intermediate molecule, which is in turn generated after punicalin degradation, and it is the immediate precursor of ellagic acid, which is the last product of ellegitannin biodegradation [42].

The results of this investigation seem to indicate that in presence of a pathogen attack, an ellagitannin biodegradation pathway is activated by fungal enzymes in the pomegranate fruit starting from punicalagin, resulting in an increased resistance to the infection, especially in the ‘Wonderful’ fruit. Indeed, several studies have described the role of pomegranate peel components as antifungal compounds. These substances are active in particular on grape berries against *Botrytis cinerea* [45], and on citrus and apple fruits against the main species belonging to *Penicillium* genus [46]. Further support to our speculation comes from the result of Belgacem et al. [47], who have evaluated the defense responses in oranges activated by pomegranate extract application in terms of relative gene expression and induction of multiple metabolic responses; in particular, the authors have observed an up-regulation of primary metabolism involved in an increased demand for energy and biosynthesis. Primary metabolites may indeed modulate the signal transduction cascades implicated in plant defense responses, causing the up-regulation of genes implicated in key pathways of secondary metabolism [47]. However, although promising, the use of plant extracts is still not common to control postharvest rots. Indeed, some issues for their large-scale use to control postharvest pathogens exist, such as the reduced or inconsistent efficacy because of fruit physiology and environment, the low residual activity and the lack of curative effect, and the limited range of activity [46].

## 4. Materials and Methods

### 4.1. Experimental Set Up

Healthy and mature pomegranate fruit, belonging to cultivars Mollar de Elche and Wonderful from two organic orchards in the same farm in Taranto province (southern Italy), were used for all trials. They were surface-sterilized by 2 min dipping in a 2% sodium hypochlorite solution, and then rinsed for 1 min in sterile distilled water. To guarantee the complete sterilization, fruit were also sprayed by 70% ethanol, and finally air-dried at room temperature. 

Inoculum was obtained using *P. granati* strain Ph1, morphologically and molecularly characterized [23] and deposited in the fungal collection of the Department of Soil, Plant and Food Sciences, University of Bari Aldo Moro (Italy).

In a first experimental set, using a cork borer (Ø 5 mm) and in aseptic conditions, three pomegranates for each cultivar were wounded at the opposite sides of the equatorial area, and inoculated by a mycelial plug (Ø 5 mm) of a 2 week old *P. granati* culture (2 plugs per fruit). Fruit inoculated by sterile PDA plugs served as a control. Each fruit was aseptically and singularly arranged in a humid chamber made by a plastic bag containing a sterile and moisturized (with 4 mL of sterile distilled water) paper towel to assure high relative humidity. All samples were incubated in the dark at 26 ± 1 °C. The incidence of decay (infected wounds, %) and the disease severity (diameter of the lesions, mm) were evaluated at 2, 4 and 6 days after pathogen inoculation.

In the second experimental set, twelve fruit for each cultivar were wounded 30-times in the equatorial area and inoculated by a mycelial plug (30 plugs/fruit), as described above. An equal number of fruits inoculated by sterile PDA plugs served as a control. Three pomegranates of both cultivars and three of the respective control fruits were sampled at 12, 24, 48 and 72 h of incubation in the conditions reported above. Using a sterile scalpel and in aseptical conditions, the infected area (110 × 40 mm) of each sample was cut off and powdered in liquid nitrogen avoiding heating up of the material. Each sample was then stored at −80 °C until use.

### 4.2. Susceptibly Assays

The ITS sequence (Genbank accession no. KU821701) of *P. granati* strain Ph1 was aligned using the free software MULTALIN (http://npsapbil.ibcp.fr/cgi-bin/npsa_automat.pl?page=/NPSA/npsa_multalin.html) with those of other species belonging to the same genus available in GenBank, i.e., *Coniella pseudogranati* (KJ869132.1), *Coniella castaneicola* (KY473972.1), *Coniella quercicola* (KX833596.1), *Coniella pseudostraminea* (KX833593.1), *Coniella straminea* (AY339348.1), *Coniella koreana* (KX833584.1), *Coniella africana* (AY339344.1), *Coniella nicotianae* (KX833590.1), *Coniella diplodiopsis* (KX833533.1), and *Coniella diplodiella* (KX833527.1). A *P. granati*-specific primer pair PILF/PILR (see above) was designed in a region characterized by deletion and SNPs using Primer3 software (http://primer3.ut.ee/) and synthesized by Macrogen (Seoul, Korea) as all the other primer pairs used in the present study. Moreover, to further confirm the genus-specificity of PILF/PILR, isolates of other fungal genera among pomegranate pathogens (*Alternaria, Penicillium, Aspergillus, Colletotrichum,* and *Botrytis*) were tested in PCR. The DNA was extracted according to Doyle and Doyle [48] and its purity and quantity were analyzed using a spectrophotometer (Nanodrop 2000, Thermo Fisher Scientific, Waltham, MA, USA) and a fluorimeter (Qubit Fluorometric Quantitation, Thermo Fisher Scientific). Reactions were arranged in a 25 μL volume using 50 ng of DNA, 0.2 μM of each primer, and 1 × Dream Taq Hot Start Green PCR Master Mix (Thermo Fischer Scientific). They were run in a T100 thermalcycler (Bio-Rad, Hercules, CA, USA) with the following conditions: 95 °C for 3 min, 35 cycles of 95 °C for 30 s, 60 °C for 30 s and 72 °C for 1 min, and 72 °C for 7 min. An amplicon aliquot (10 µL) was run on 1.5% agarose gel in TBE buffer (1×), pre-stained with GelRed^®^ (Biotium, Landing Parkway Fremont, CA, USA), and visualized by Gel Doc^™^ EZ System (Bio-Rad).

To evaluate the specificity and sensitivity of PILF/PILR primer pair, a calibration curve was built up in qPCR. Reactions were carried out in triplicate in 96 wells PCR plates, in a CFX96 Touch Real-time PCR Detection System (Bio-Rad). The 20-μL reaction mixtures contained 1× PowerUp SYBR Green (Thermo Fisher Scientific), 0.2 μM of each primer, and 1 μL of ten-fold genomic DNA dilutions (from 3.9 to 3.9 × 10^−5^ ng) as the template; in the negative control reactions, water replaced template DNA. Cycling conditions were set as follows: 50 °C for 2 min, 95 °C for 2 min, followed by 45 cycles at 95 °C for 3 s and 60 °C for 30 s. To confirm assay specificity, melting curves were obtained at temperatures ranging from 65 to 95 °C. Acquisition was performed every 0.5 °C increase in temperature, with a 10 s step. Moreover, an amplicon aliquot (10 µL) was run on 1.5% agarose gel. The R^2^, PCR efficiency, and linear equation were obtained using the instrument associate software by plotting Ct values (*y*-axis) against logs of DNA (ng, *x*-axis). This curve was used as a reference standard to extrapolate quantitative information for DNA targets of unknown concentrations. Then, for each time point (12–72 h) and cultivar (Mollar de Elche and Wonderful) for both inoculated and non-inoculated samples, the genomic DNA was extracted from 75 mg of powdered tissue using the Plant/Fungi DNA Isolation Kit (Norgen Biotek Corp., Thorold, ON, Canada) according to manufacturer’s recommendation. The quantity and quality of each sample were analyzed as described above, and then samples were tested in qPCR. Briefly, 1 μL of each genomic DNA was examined in triplicate arranging reaction mixtures and qPCR amplification conditions as reported above.

To take into account variations in sample size (weight of tissue used for the DNA extraction) and in efficiency of extraction and amplification, the actual concentration of *P. granati* DNA in each sample was expressed as normalized data according to the quantity of host DNA (SQ). Thus, a primer pair targeting the sequence (KU977461.1) of EF-1α gene available in GenBank was designed (Table 1) and used as the internal control. Uncorrected *P. granati* DNA concentrations were multiplied by a correction factor calculated as follows: average host DNA concentration/host DNA concentration of the specific sample under investigation [32].

### 4.3. Gene Expression Assays

Total RNA was extracted from 75 mg of powdered tissue using the Plant/Fungi Total RNA Purification Kit (Norgen Biotek Corp.), modified by adding 2.5% of PVP-40 (Sigma-Aldrich, Merck KGaA, Darmstadt, Germany) to the Lysis Buffer, and inserting an intermediate washing step by 2-butoxyethanol (400 μL) (Sigma-Aldrich). Furthermore, the total RNA was treated by the RNase-Free DNase I Kit (Norgen Biotek Corp.) to avoid any DNA contamination. RNA yield and purity were assessed as reported above for DNA, plus electrophoresis on 1.5% agarose gel. RNA samples were stored at −80 °C until use. Each total RNA sample (100 ng) was reverse-transcribed using the SuperScript IV VILO Master Mix (Thermo Fisher Scientific), according to manufacturer’s recommendations in a T100 thermalcycler (Bio-Rad).

Three genes known to be involved in the host resistance and the constitutively expressed housekeeping gene EF-1α were targeted (Table 1). Both sequences of PAL (KY094504.2; KY433997.1) and of class III chitinase (KU977459.1; AB605773.1) genes available in GenBank were aligned and specific primer pairs were designed on conserved portions. Similarly, a specific primer pair was designed on the single sequences of peroxidase (KY129694.1) available in GenBank. Furthermore, five serial dilutions (from 10^−2^ to 10^3^ ng) of RNA were used to evaluate, for each gene, the range of concentrations in which target RNA and Ct values were linearly correlated and to determine the reaction efficiency. The cDNA was synthesized from each dilution as reported above. The qPCR reactions contained 2 μL of cDNA, 0.2 μM of each primer and 1 × PowerUp SYBR Green Master Mix (Thermo Fisher Scientific), according to manufacturer’s recommendation; each reaction was run in triplicate in a CFX96 Touch Real-time PCR Detection System (Bio-Rad). Cycling and melting curve conditions were the same used for DNA amplification. 

The relative expression of PAL, peroxidase, and chitinase genes was evaluated according to ΔΔCt method [49]. Values were automatically generated by the Bio-Rad instrumental software and normalized according to the EF-1α gene. Data were reported as fold relative expression as compared to the wounded non inoculated control and transformed to log_2_. In particular, the level of change (i.e., either increase or decrease) in gene expression was categorized on the basis of the following range in log_2_ transformed ratios: “low” ≥−1.0 to ≤1.0; “medium” ≥−2.0 to <−1.0, or >1.0 to ≤2.0; “high” <−2.0, or >2.0 [50].

### 4.4. Enzymatic Assays

From each sample and sampling time, 10 g of powdered pomegranate tissue were homogenized with 50 mM sodium acetate buffer pH 5.6 (1:1, *w*/*v*), and centrifuged (15 min at 10,000 × *g* and 4 °C). The supernatant was vacuum filtered through filter paper by a Buchner funnel. Filtered juices were added to 60% acetone (*v*/*v*) and incubated at −20 °C for 2 h to precipitate proteins. Samples were centrifuged (30 min at 10,000× *g* and 4 °C), and each resulting pellet was washed three times with 60% refrigerated acetone. Pellets were air-dried and re-suspended in 2 mL of 50 mM sodium acetate buffer (pH 5.6), crushing the pellet with a pestle. Protein extracts were stored at −20 °C until use. The protein concentration was determined according to Bradford [51] with the Quick Start^™^ Bradford Protein Assay (Bio-Rad) and expressed as mg/L.

Chitinase activity was tested using dye-labeled carboxymethyl chitin-RBV (Loewe Biochemica GmbH, Germany), according to Wirth and Wolf [52] protocol. Briefly, 100 μL of each protein extract, 100 μL of CM chitin-RBV and 200 μL of 50 mM phosphate buffer (pH 6.4) were mixed and incubated at 37 °C for 2 h; reactions were stopped in ice adding 100 μL of 2 N HCl. Samples were centrifuged (10,000 × *g*) and the absorbance of the supernatant was measured at 550 nm (Multiskan EX, Labsystem, Finland). Chitinase specific activity was expressed in U/μg/s on a total protein basis.

Guaiacol was used as the substrate to evaluate peroxidase activity [53]. The reaction mixture, consisting of 100 μL of crude extract and 100 μL of 50 mM sodium acetate buffer pH 5.6 amended with 10 mM of guaiacol (Sigma-Aldrich) and 10 mM H_2_O_2_, (Sigma-Aldrich), was incubated for 60 s at room temperature. The absorbance at 470 nm was spectrophotometrically measured (Beckman DU 640 Spectrophotometer, Corona, CA, USA); the peroxidase specific activity was expressed as U/μg/s on a total protein basis.

To measure PAL activity, 100 μL of crude extract and 100 μL of 0.1 M L-phenylalanine (Sigma-Aldrich) were added in 0.1 M borate buffer (pH 8.8), according to Beaudoin-Eagan and Thorpe [54] with some modifications. Samples were incubated at 30 °C for 2 h. The reaction was then stopped by adding 100 μL of 6 N HCl and cooling in ice for 5 min. Samples were then centrifuged at 10,000 × *g* for 5 min. The amount of cinnamic acid produced was evaluated spectrophotometrically (Beckman DU 640 Spectrophotometer) at 290 nm; PAL specific activity was expressed as ng/μg/h of cinnamic acid on a total protein basis.

β-1,3-Glucanase activity was estimated following the Abeles and Forrence [55] protocol: 62.5 μL of protein extract and 62.5 μL of 4 % (*w*/*v*) laminarin (Sigma-Aldrich) were incubated at 37 °C for 2 h. Every reaction was stopped by adding 375 μL of 3,5-dinitrosalicylic acid (DNS) (Sigma-Aldrich) and by heating in boiling water for 10 min, followed by rapidly cooling in ice. The absorbance was measured at 492 nm (Multiskan EX, Labsystem); glucanase specific activity was reported as µmol/µg/min glucose on a protein mass basis.

Enzymatic assays were arranged in triplicate and average values were obtained.

### 4.5. Chemical Analysis of Phenolics

The same powdered material was used to extract total phenolics and to detect the qualitative HPLC-UV/DAD profile of pomegranate rinds. In addition, the most significant samples were characterized by HPLC-ESI-MS and MS^2^. All the procedures were carried out in duplicate according to Brighenti et al. [40]. All reagents were of chromatographic grade.

Briefly, 0.4 g of powdered tissue was added to 10 mL of a mixture of H_2_O and ethanol 80:20 (*v*/*v*) with 0.1% HCl as the extraction solvent for the maceration; these were mixed at room temperature for 30 min, under magnetic stirring. Then mixtures were centrifuged at 1162× *g* for 5 min and the supernatant solution was vacuum filtered in a volumetric flask. To increase the yield of maceration, residues of the early extraction were re-extracted as above. Filtrates of both extractions were combined and diluted to 25 mL using the same solvent. 

These extracts were used to quantify total phenolics, according to the Folin–Ciocalteu colorimetric assay [56]. Serial dilutions of gallic acid stock solution were arranged to develop a calibration curve. Then 500 μL of Folin–Ciocalteu reagent (Sigma-Aldrich) and 1 mL of a saturated and filtered solution of NaCO_3_ were added to 50 μL of both gallic acid standard solution and pomegranate extracts in a 10 mL volumetric flask, reaching the final volume with distilled water. The resulting solutions were gently mixed and incubated at room temperature in the dark for 2 h before the absorbance was measured at 765 nm spectrophotometrically (Beckman DU 640 Spectrophotometer). By interpolating the absorbance values of the unknown samples in the GA calibration curve, the total phenolic content of every sample was found out, which was expressed as mg/g GAE.

As regards the HPLC analysis, the pomegranate rind extracts obtained above were filtered using a 0.45 μm PTFE filter into a vial and injected into the HPLC system. The equipment used in this work was an Agilent Technologies (Waldbronn, Germany) modular model 1100 system, consisting of a vacuum degasser, a quaternary pump, an auto-sampler, a thermostated column compartment and a diode array detector (UV/DAD). The chromatograms were recorded by using an Agilent Chemstation for LC and LC–MS systems (Rev. B.01.03). The analyses were carried out on an Ascentis Express C_18_ column (150 × 3.0 mm I.D., 2.7 m, Supelco, Bellefonte, PA, USA). The mobile phase was composed of (A) 2% HCOOH in H_2_O and (B) 0.5% HCOOH in MeOH-H_2_O (9:1, *v*/*v*). The separation was achieved by using a gradient elution as follows: 0–13 min 2% B, 13–18 min from 2% to 5% B, 18–23 min from 5% to 10% B, 23–43 min from 10% to 25% B, 43–53 min from 25% to 50% B, 53–58 min from 50% to 100% B, 58–68 min 100% B, 68–71 min from 100% to 2% B. The post-running time was 5 min. The flow-rate was 0.4 mL/min. The column temperature was set at 30 °C. The sample injection volume was 3 μL. The UV/DAD acquisitions were carried out in the range 190–600 nm and chromatograms were acquired at 268, 310 and 520 nm. Three injections were performed for each sample.

The HPLC-ESI-MS and MS^2^ analyses were performed by using an Agilent Technologies modular 1200 system, equipped with a vacuum degasser, a binary pump, thermostated autosampler and column, plus a 6310A ion trap mass analyzer with an ESI ion source. The HPLC column and the applied chromatographic conditions were the same as those used for the HPLC-UV/DAD system. The HPLC-ESI-MS system operated both in the positive and in the negative ion mode, by performing a run for each polarity. For the positive ion mode, the experimental parameters were set as follows: the capillary voltage was 3.5 kV, the nebulizer (N_2_) pressure was 32 psi, the drying gas temperature was 350 °C, the drying gas flow was 10 L/min and the skimmer voltage was 40 V. For the negative ion mode, the MS conditions were the same as described above, with the exception of the capillary voltage that was set at 4.0 kV. Data were acquired by Agilent 6300 Series Ion Trap LC/MS system software (version 6.2). The mass spectrometer was operated in the full-scan mode in the *m*/*z* range 100–1500. MS^2^ spectra were automatically performed with helium as the collision gas in the *m*/*z* range 50–1500, by using the SmartFrag function. 

### 4.6. Data Analysis

The difference in the ability to cause the disease was calculated with the following formula: (X−Y)/X × 100, where X and Y correspond to the mean percentage of infected wounds or lesion diameter in the two cultivars, respectively.

Using the statistical software package Statistics for Windows (StatSoft, Tulsa, OK, USA), data were subjected to ANOVA (one-way analysis of variance). Percentage data of incidence of decay were arcsine-square root transformed before ANOVA analysis. If statistical analysis determined homogeneity of variances, data from repeated experiments were combined. Significant differences (*p* < 0.05) were identified by the General Linear Model (GLM) procedure using the DMRT.

## 5. Conclusions

In the present investigation, mature pomegranate fruit from organic agriculture of southern Italy of two of the most widespread cultivars were compared for their susceptibility to *P. granati* that is considered a destructive pathogen. The cultivar Mollar de Elche was found to be more susceptible than ‘Wonderful’. Indeed, it showed a major growth of the pathogen, as confirmed by the diameter of the lesions and the specific qPCR assay, which detected a higher amount of pathogen DNA in pomegranate rind tissue of the cultivar Mollar de Elche. Comparing the relative expression of PAL, peroxidase, and chitinase genes between the two cultivars, ‘Wonderful’ displayed a stronger up-regulation, especially starting from 48 h post inoculation. Biochemical data were in good agreement with gene expression assays. As a consequence, fruit from cultivar Wonderful should be more prompt to a defensive response. Furthermore, the chromatographic profile of pomegranate rind extracts showed a shift in the amount and composition of phenolic compounds, especially in cultivar Wonderful. Indeed, the HPLC analysis of the extracts indicated the presence of characteristic peaks in the inoculated samples of the two cultivars, starting from 48 h post-inoculation, corresponding to phenolic compounds coming from the degradation of punicalagin, which is the main pomegranate ellagitannin. Since *P. granati* is able to lysate this class of compounds by its enzymatic activity, it is reasonable that ‘Wonderful’ enhances the phenolic production as a response to the pathogen attack, and the fungus hydrolyses them in a positive feedback mechanism that ends up in a minor susceptibility of this variety. Although these findings need to be further confirmed in further samples coming from the same cultivars under different latitudes and sampling cases (orchards, agronomical conditions, harvest times, etc.), they seem to indicate that polyphenols might have a significance in the different susceptibility of the two pomegranate cultivars to *P. granati,* and thus their content might constitute a putative marker in selecting programs of new resistant germplasm.

## Figures and Tables

**Table 1:** Gene, primer name and sequence (5′-3′), amplicon size (bp), linear equations, R^2^ and reaction efficiencies (%) obtained by plotting RNA concentrations (log ng), and Ct values experimentally achieved by qPCR for EF-1α, chitinase, PAL, and peroxidase genes.

Gene	Primer	Sequence	Amplicon Size	Linear Equation	R^2^	Reaction Efficiency
EF-1α	EF-F EF-R	ATGATTCCCACCAAGCCCAT GGGTCCTTCTTCTCCACACT	128	y = −3.34x + 25.84	0.999	99.2
Chitinase	CHIT-F CHIT-R	GCCTGAGCGACGAAATAAGG CAGGTAATCGGCGAAATTGT	125	y = –3.24x + 22.05	0.995	103.5
PAL	PAL2-F PAL2-R	GCAATCGGGAAGCTGATGTTT TCMGAGCAGTATGAGGCCAT	152	y = −3.50x + 30.98	0.995	93.0
Peroxidase	POD1-F POD1-R	CCCCGCTGTACAAGTTCCTA TGAAGTTGTGGGCGCATAAC	128	y = −3.53x + 41.95	0.974	92.0

**Table 2:** Retention times and MS data of phenolic compounds detected in pomegranate rind. Experimental conditions as in Materials and Methods section. The MS^2^ data were obtained from the fragmentation of the precursor ion marked with an asterisk. Relative intensities of product ions are in parentheses.

Peak Number	Compound Name	*t*_R_ (min)	Precursor Ions (*m/z*)	Product Ions (*m/z*)
1	Gallagic acid	2.0	601 [M − H]^−^ *	-
2	HHDP-glucose	2.0	481 [M − H]^−^ *	301 (100)
3	Punicalin	5.6	781 [M − H]^−^ *	721 (21), 601 (100)
4	α-Punicalagin	13.7	1083 [M − H]^−^ * 541 [M – 2H]^2–^	781 (32), 721 (15), 601 (100), 575 (14)
5	β-Punicalagin	26.8	1083 [M − H]^−^ * 541 [M – 2H]^2–^	781 (46), 721 (13), 601 (100), 575 (16)
6	Ellagic acid hexoside	48.2	463 [M − H]^−^ *	301 (100)
7	Ellagic acid pentoside	53.3	433 [M − H]^−^ *	301 (64), 300 (100)
8	Ellagic acid deoxyhexoside	53.6	447 [M − H]^−^ *	301 (77), 300 (100)
9	Ellagic acid	58.0	301 [M − H]^−^ *	-

* precursor ion.
